# SARS-CoV-2 PCR and antibody positivity among school staff at the beginning and end of the first school term

**DOI:** 10.1186/s12889-021-12134-4

**Published:** 2021-11-11

**Authors:** Moza Alishaq, Andrew Jeremijenko, Hanaa Nafady-Hego, Jameela Ali Al Ajmi, Mohamed Elgendy, Anil George Thomas, Peter V. Coyle, Hamed Elgendy, Abdul-Badi Abou-Samra, Adeel A. Butt

**Affiliations:** 1 Hamad Medical Corporation, PO Box 3050, Doha, Qatar; 2 Microbiology and Immunology Department, Faculty of Medicine, Assiut University, Assiut, Egypt; 3 Faculty of Medicine, Universiti Sains of Malaysia, Kota Bharu, Kelantan Malaysia; 4 Department of Medicine and Department of Population Health Sciences, Weill Cornell Medical College, New York, NY USA; 5 Anesthesia Department, Faculty of Medicine, Assiut University, Assiut, Egypt; 6 Weill Cornell Medicine - Qatar, Doha, Qatar

**Keywords:** COVID-19, SARS-CoV-2, Qatar, Schools

## Abstract

**Background:**

There is controversy regarding the role of in-person attendance in schools and transmission of the SARS-CoV-2 pandemic. Several studies have demonstrated no increase in transmission, while some have reported large outbreaks with in-person attendance. We determined the incidence and risk factors for SARS-CoV-2 infection among school staff after one school term.

**Methods:**

Nasopharyngeal swabs (NPS) for SARS-CoV-2 RT-PCR and blood for SARS-CoV-2 antibody testing were obtained from staff at a large international school in Qatar at the beginning of the 2020–2021 school year and repeated at the end of the first term.

**Results:**

A total of 376 staff provided samples for testing. At the beginning of the 2020–2021 school year, the PCR positivity for SARS-CoV-2 was 13%, while seropositivity was 30.1%. A majority of those who tested positive either by PCR or serologically, were non-teaching staff. At the end of the first school term four months later, only 3.5% of the initially antibody-negative staff had seroconverted. In multivariable logistic regression analysis, male gender (OR 11.48, 95%CI 4.77–27.64), non-teaching job category (OR 3.09, 95%CI 1.10–8.64), contact with a confirmed case (OR 20.81, 95%CI 2.90–149.18), and presence of symptoms in the preceding 2 weeks [1–2 symptoms OR 4.82, 95%CI 1.79–12.94); ≥3 symptoms OR 42.30, 95%CI 3.76–476.43) independently predicted SARS-CoV-2 infection in school staff before school starting.

**Conclusion:**

Male gender, non-teaching job, presence of symptoms, and exposure to a confirmed case were associated with higher risk of infection. These data can help policymakers in determining the optimal strategy for school reopening.

## Background

There is significant controversy regarding the timing of reopening schools with full in-person attendance in the Severe Acute Respiratory Syndrome Coronavirus 2 (SARS-CoV-2) pandemic era. Proponents of early opening with full in-person attendance cite the benefits of human interaction upon the psychological and academic growth of children and the enormous physical, psychological, and financial burden placed on the families as reasons to allow immediate opening with full in-person attendance [1, 2]. Opponents cite the ongoing pandemic with increasing number of cases, hospitalizations and mortality in infected persons and the possible enhanced role of children in propagation of the pandemic as reasons to delay in-person attendance indefinitely [3, 4]. Understanding the transmission dynamics in schools can help policy makers make informed decisions. Several studies from the United States and Europe have shown no increase in transmission of SARS-CoV-2 infection associated with in-person attendance or school opening [5, 6]. Conversely, rare large outbreaks with apparent transmission in schools have also been reported [7]. While overall evidence suggests little transmission within schools, controversy still exists regarding the role of schools and in-person attendance with spread of infection. Seroprevalence studies over time have suggested low levels on within-school transmission, with majority of the infections acquired outside the school settings [8]. Within the schools, in-person sporting activities have been associated with a higher risk of transmission [9]. Due to such conflicting evidence, specific recommendations regarding the timing of school opening, and the need for distance or blended learning as opposed to in-person learning are lacking. We undertook this study to define the baseline prevalence of SARS-CoV-2 infection and incidence of infection after one full term of school among the staff of an international school in Qatar.

## Methods

### Study population and participants

As a part of the national testing and screening drive, staff at multiple schools in Qatar provided a nasopharyngeal swab (NPS) to test for the SARS-CoV-2 by real-time reverse transcription polymerase chain reaction (RT-PCR). The 2020–2021 school year in Qatar started on August 25, 2020 with the term ending in the middle of December 2020. A series of measures were implemented in all schools (including the one chosen for the current study) to reduce the likelihood of infection transmission. These included a requirement for all staff to have a negative NPS for SARS-CoV-2 by PCR at the beginning of the school year, hybrid learning for student with maximum 30% attendance on any given day on a rotational basis, and strict mask use and physical distancing policies for all staff, students and attendants dropping off or picking up students. Based on convenient access, large number of staff, and widespread willingness to participate in the national screening program, we chose one school for analysis. During the study period, the school instituted reduced capacity (< 30% at any given time), temperature screening of all attendees, display of hand hygiene posters and extensive distribution of hand sanitizers.

### Data collection

As a part of the national surveillance campaign, baseline nasopharyngeal swab for SARS-CoV-2 PCR and blood for SARS-CoV-2 antibody testing at the beginning of the 2020–2021 school year were obtained on all available staff. Repeat testing at the end of the first term was offered to all staff as a part of the ongoing follow-up. All testing was performed at a single national reference laboratory using validated commercial assays as described in our previous studies and detailed below [10, 11]. A brief structured interview was administered to all participants to determine presence of symptoms, accommodation type, exposure to any confirmed cases, and presence of comorbidities. The data collected for above purposes was subsequently secondarily analyzed for the current study purposes.

### Laboratory methods

Roche Elecsys® Anti-SARS-CoV-2 (99.5% sensitivity, 99.8% specificity, an electrochemiluminescence immunoassay, was used for antibody detection in the serological samples. Result interpretation followed manufacturer instructions: reactive for optical density (a proxy for antibody titer) cutoff index ≥1.0 and non-reactive for cutoff index < 1.0 [12, 13].

Nasopharyngeal and/or oropharyngeal swabs were collected for RT-PCR testing and placed in Universal Transport Medium (UTM). Aliquots of UTM were: extracted on a QIAsymphony platform (QIAGEN, USA) and tested with real-time reverse-transcription PCR (RT-qPCR) using TaqPath™ COVID-19 Combo Kits (Thermo Fisher Scientific, USA) on an ABI 7500 FAST (Thermo Fisher, USA); tested directly on the Cepheid GeneXpert system using the Xpert Xpress SARS-CoV-2 (Cepheid, USA); or loaded directly into a Roche cobas® 6800 system and assayed with a cobas® SARS-CoV-2 Test (Roche, Switzerland). The first assay targets the viral S, N, and ORF1ab gene regions. The second targets the viral N and E-gene regions, and the third targets the ORF1ab and E-gene regions. All RT-PCR testing was conducted at the Hamad Medical Corporation Central Laboratory following standardized protocols.

### Statistical analysis

Quantitative data presented as median and IQR (interquartile range) and Mann-Whitney U test was used to compare the study groups. Qualitative data presented in the form of frequency and percentage and Chi-square test was used to compare between the categorical data. Wilcoxon’s test was used to compare anti SARS Cov-2 antibody titer before and after school opening. A logistic regression model was used to calculate the odds ratios and 95% confidence intervals for factors associated with infection (PCR or antibody positivity). Factors that showed a *p*-value of ≤0.05 were included in the multivariable model. Statistical significance was set at *P* < 0.05. The data were analyzed using Statistical Package of Social Sciences (IBM-SPSS 21), (SPSS: An IBM Company, version 21.0, IBM Corporation, Armonk, NY, USA).

The study was approved by the Institutional Review Board at Hamad Medical Corporation (approval number MRC-01-20-982). A waiver of informed consent was granted since study procedures were carried out as part of the national pandemic response.

## Results

A total of 376 staff provided a nasopharyngeal swab for PCR testing and a blood sample for serologic testing for SARS-CoV-2 at the beginning of the school year. The median age (IQR [interquartile range]) was 42 years (IQR 36, 49), 43.6% were males and 56.6% were teaching staff (Table 1). At the beginning of the school year, only 3.8% gave a history of exposure to a person with confirmed SARS-CoV-2 infection. Overall, 54.8% lived in a single family housing unit, 44.1% in shared accommodation and 1.1% lived alone in single-person accommodations.

**Table 1:** Baseline characteristics of the teaching and non-teaching school staff

	Total *N* = 376	Teaching staff *N* = 213	Non-teaching staff *N* = 163	*p*-value
**Median age (IQR), years**	42 (36, 49)	41 (36, 47)	43 (36, 51)	0.09
**Male sex, N (%)**	165 (43.6)	39 (18.3)	126 (77.3)	< 0.0001
**Nationality, N (%)**				< 0.0001
Indian	287 (76.3)	206 (96.7)	81 (49.7)	
Nepalese	63 (16.8)	1 (0.5)	62 (38.0)	
Sri Lankan	15 (4.0)	2 (0.9)	13 (8.0)	
Bangladeshi	4 (1.1)	1 (0.5)	3 (1.8)	
Filipino	2 (0.5)	0	2 (1.2)	
Kenyan	2 (0.5)	0	2 (1.2)	
Others	3 (0.8)	3 (1.4)	0	
**Type of accommodation, N (%)**				< 0.0001
Single	4 (1.1)	3 (1.4)	1 (0.6)	
Shared	166 (44.1)	32 (15)	134 (82.2)	
Family	206 (54.8)	178 (83.6)	28 (17.2)	
**Job category, N (%)**				< 0.0001
Teachers	213 (56.6)	213 (100)	N/A	
Administrative and support staff	70 (18.6)	N/A	70 (42.9)	
Transportation and security staff	58 (15.4)	N/A	58 (35.6)	
Technician/conductor/laboratory workers	21 (5.6)	N/A	21 (12.9)	
Service staff	14 (3.7)	N/A	14 (8.6)	
**Median Education years (IQR)**	16 (10, 16)	16 (16,16)	10 (9,12)	< 0.0001
PCR positive	49 (13.0%)	8 (16.3%)	41 (83.7%)	< 0.0001
Median (IQR) Ct Values	23.8 (18.2, 30.0)	19.7 (17.1, 33.2)	23.8 (18.3, 30.0)	
Antibody positive	113 (30.1%)	19 (16.8%)	94 (83.2%)	< 0.0001
Median (IQR) antibody titer	61.3 (18.0, 101.0)	60.7 (16.6, 96.5)	61.5 (18.3, 101.2)	
History of contact with confirmed cases	14 (3.8%)	5 (35.7%)	9 (64.3%)	0.11
Presence of clinical symptoms	113 (30.1%)	88 (77.9%)	25 (22.1%)	0.03
Presence of Co morbidities	32 (8.5%)	17 (53.1%)	15 (46.9%)	0.69

At the beginning of the school year, 49 (13.0%) were PCR-positive; 8 (16.3%) of them were teachers, while 113 (30.1%) were seropositive for SARS-CoV-2; 19 (16.8%) were teachers. Nearly one-third (30.1%) reported having at least one symptom compatible with SARS-CoV-2 infection in the 2 weeks preceding the testing. Only 14 (3.8%) reported history of contact with a person with confirmed SARS-CoV-2 infection (Table 1).

**Fig. 1:**
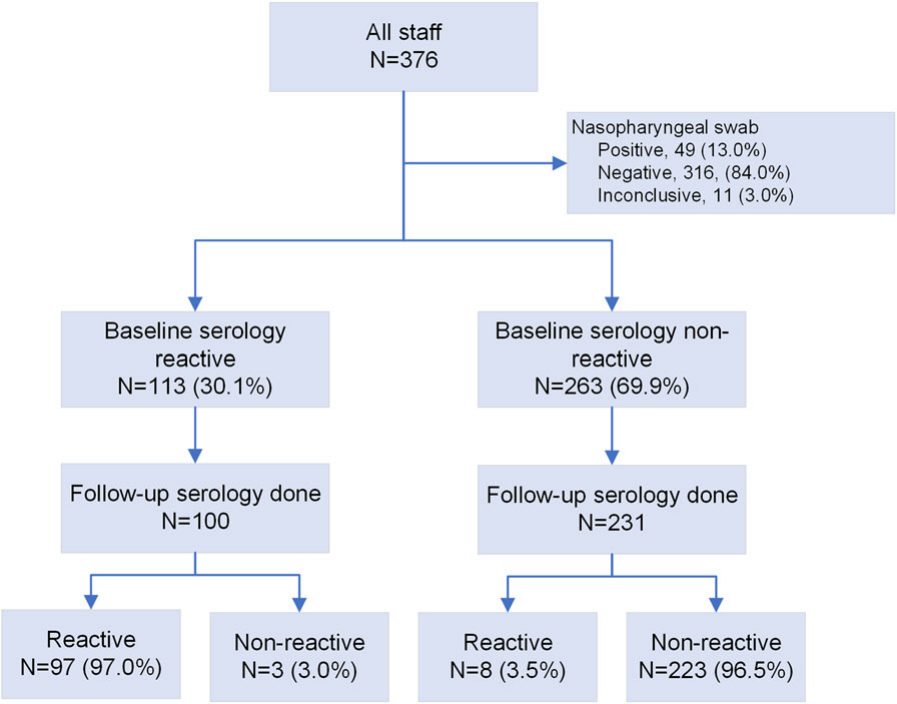
Baseline and follow-up serologic testing among all school staff

At baseline, 113 (30.1%) were seropositive for SARS-CoV-2 antibody. Follow-up serology at the end of the first school term was available for 100 of these staff (median time to re-testing 17.1, IQR 17.1, 17.4 weeks) with 97 (97.0%) remaining seropositive. Of the 263 staff with a negative serology at baseline, 231 were retested at the end of the first term (median 17.3 weeks, IQR 17.1, 17.4 weeks) and 8 (3.5%) were seropositive (Fig. 1). Of the seroconverted 7 (87.5%) were teachers and 6 of them had PCR positive results. Anti SARS Cov-2 antibody titer significantly reduced after school opening in non-teaching staff (median antibody before and after school opening: 61.5 (IQR: 18.28, 101.25), and 30 (IQR: 8.4, 70.8) respectively; *P* < 0.0001). The change in teaching staff before and after school opening was not significant. (median antibody titer before and after school opening: 60.7 (IQR: 16.6, 96.5), and 47.7 (IQR: 23.6, 106.6) respectively; *p* = 0.87).

In multivariable logistic regression analysis, male gender (OR 11.48, 95%CI 4.77–27.64, *p* < 0.0001), non-teaching profession (OR 3.09, 95% CI 1.10–8.64, *p* = 0.03) contact with a confirmed case (OR 20.81, 95% CI 2.90–149.18, *p* = 0.003), and presence of symptoms in the preceding 2 weeks [1–2 symptoms (OR 4.82, 95% CI 1.79–12.9), (3 or more symptoms (OR 42.30, 95% CI 3.76–476.43,) *p* = 0.002)] independently predicted SARS-CoV-2 infection (PCR or antibody positive) in school staff before school starting (Table 2).

**Table 2:** Factors associated with SARS-CoV-2 infection at the initial visit in school staff (PCR positive and/or antibody positive; multivariable logistic regression analysis)

	Odds ratio (OR)	95% CI	*p*-value
Age/years	1.008	(0.974–1.043)	0.6
Male gender	11.48	(4.771–27.641)	< 0.0001
Education/years	0.90	(0.797–1.025)	0.1
Non-teaching staff (vs. teaching staff)	3.09	(1.102–8.638)	0.03
Shared accommodation	2.25	(.899–5.654)	0.08
History of contact with confirmed cases	20.81	(2.904–149.184)	0.003
Symptoms in the 2 weeks prior to testing			
None	Ref.		
1–2	4.82	(1.792–12.94)	0.002
3 or more	42.30	(3.756–476.432)	0.002
Co morbidities			
None	Ref.		
1–2	1.05	(0.26–4.213)	0.9
3 or more	3.70	(0.312–43.78)	0.3

## Discussion

Our study provides critical insights into the SARS-CoV-2 infection dynamics among the staff of a large school which can help inform policymakers make informed decisions on school openings.

At the beginning of the school year, 13% of the staff were positive by PCR on a NPS sample and therefore likely infectious. Since every staff member was required to get tested before resuming duty, this represents a true infection rate. At that point in time, approximately 5% of Qatar’s population had been diagnosed with SARS-CoV-2 infection [10]. However, the national positivity rate reflects only those who were tested and is not a true reflection of infection prevalence in the country. Indeed, in certain population subgroups in Qatar, infection rates in excess of 50% had been reported [11]. Importantly, the requirement to test every staff member prior to resuming duty removed potentially infectious persons from the school environment thereby reducing the risk of transmission to students and coworkers.

At the beginning of the school year, the seroprevalence among the staff was 30%. This implies that a large proportion of persons were not diagnosed at the time of acute infection when their potential to transmit infection is the highest. Antibody testing is not a robust strategy to reduce the transmission of infection since antibody response lags the infectious period by days to weeks. Antibody testing may be helpful in identifying staff with prior infection who may now be immune to reinfection and therefore unlikely to transmit infection. Whether persons with prior natural infection and spontaneous recovery may still harbor the virus and potentially transmit it is not entirely clear. Of the 113 seropositive persons at baseline in our study, 49 (43%) also had a positive NPS by PCR. One possible explanation is that these persons were recently infected and were in the overlap period between complete viral clearance and early antibody response.

The prevalence of acute infection (PCR positivity) and seropositivity at baseline were lower among teaching staff compared with the non-teaching staff. This is likely due to the majority of teaching staff living in a single family setting while the majority of the non-teaching staff were living in shared accommodations with non-related room-mates. In other studies from Qatar, the infection rate among craft and manual workers sharing accommodations has been shown to be much higher than the population living in family accommodations [11, 14]. History of contact with a confirmed infected case, male gender, sharing accommodations, and presence of symptoms were strongly associated with infection before the school opening. These can explain the lower prevalence of SARS-CoV-2 infection among teachers, since a majority of the teachers were females, living in family accommodations and the sample timing was 5 months after school closure in Qatar. Male school staff are predominantly in the transportation department (school bus drivers, conductors) and security. They also tend to be overwhelmingly single and live in shared accommodation with non-family members. Hence, their exposure is more than female staff, who are predominantly teachers, who are married and liver with their own family. We previously reported the strong correlation between shared accommodation and viral infection [15]. These results suggests that teachers pose a low risk of infection transmission, and if a decision to reopen schools were to be made, a targeted screening of non-teaching staff may be a useful strategy to reduce transmission. Previously published reports from other countries support our results. In a study from Germany that included 177 primary and 175 secondary school students, 142 staff and 625 household members, prevalence of SARS-CoV-2 infection was 2.7% among students, 1.4% among staff, and 2.3% among household members. Contact with confirmed cases outside the school was associated with infection among study participants [16]. Low attack rates within schools were also reported from a large study from North Carolina, USA, which included over 100,000 staff and students from 13 school districts [9]. An Italian study did not find any increase in infection incidence among students who attended in-person classes compared with those who attended remote learning [8]. On the other hand, breakdown in infection control practices, e.g. not wearing a mask especially by unvaccinated teachers or staff, has been associated with large outbreak among their students [17].

A limitation of our study is that it was conducted at a single school and may not be representative of the entire school staff population in the country, or indeed rest of the world. However, repeat testing on the same population before and after the opening of the school provides an accurate and clear picture of infection dynamics. Another limitation is that we did not assess the infection rates among students. Based on national surveillance data, there was no spike in infection rates among children of school-going age during that period. However, this population is not routinely screened so firm conclusions cannot be drawn.

## Conclusion

Our study provides data that can be used to design a logical testing and screening strategy for school staff, and to devise an evidence-based strategy to reopen in-person learning in a safe manner. Based on our data and prior literature on this subject, teachers are less likely to transmit infection to students if a strict vaccination and masking policy is enforced. Non-teaching staff may pose a higher risk due to their more frequent exposure risk. Targeted screening strategies can be developed to mitigate the risk of infection transmission in schools.

## Data Availability

The datasets used and/or analysed during the current study available from the corresponding author on reasonable request.

## Abbreviations

COVID-19: Coronavirus disease
IQR: interquartile range
NPS: Nasopharyngeal swabs
OR: Odds ratio
RT-PCR: Real-Time- Polymerase Chain Reaction
SARS-CoV-2: Severe acute respiratory syndrome coronavirus 2
